# The Genetic Makeup of Myeloproliferative Neoplasms: Role of Germline Variants in Defining Disease Risk, Phenotypic Diversity and Outcome

**DOI:** 10.3390/cells10102597

**Published:** 2021-09-29

**Authors:** Elena Masselli, Giulia Pozzi, Cecilia Carubbi, Marco Vitale

**Affiliations:** 1 Department of Medicine and Surgery, Anatomy Unit, University of Parma, 43126 Parma, Italy; elena.masselli@unipr.it (E.M.); giulia.pozzi@unipr.it (G.P.); 2 University Hospital of Parma, AOU-PR, 43126 Parma, Italy

**Keywords:** myeloproliferative neoplasms, polymorphism, GWAS, germline predisposition, inflammation, clonal hematopoiesis

## Abstract

Myeloproliferative neoplasms are hematologic malignancies typified by a substantial heritable component. Germline variants may affect the risk of developing a MPN, as documented by GWAS studies on large patient cohorts. In addition, once the MPN occurred, inherited host genetic factors can be responsible for tuning the disease phenotypic presentation, outcome, and response to therapy. This review covered the polymorphisms that have been variably associated to MPNs, discussing them in the functional perspective of the biological pathways involved. Finally, we reviewed host genetic determinants of clonal hematopoiesis, a pre-malignant state that may anticipate overt hematologic neoplasms including MPNs.

## 1. Introduction

Classical Philadelphia chromosome-negative myeloproliferative neoplasms (MPNs) are a group of closely related stem cell disorders, namely, polycythemia vera (PV), essential thrombocythemia (ET), and pre-fibrotic and overt primary myelofibrosis (prePMF and overtPMF, respectively). Myelofibrosis may also evolve from an antecedent PV or ET, therefore termed as post-PV or post-ET MF. Bone marrow myeloid lineage(s) expansion, coupled with a variable degree of reticulin/collage fiber deposition, abnormal peripheral blood cell count, extramedullary hematopoiesis, organomegaly, and increased inflammatory burden are hallmarks of MPNs [1].

Clonal proliferation is triggered by the acquisition of somatic mutations in specific myeloid genes, operationally classified in *driver* mutations, e.g., *JAK2*V617F and *JAK2* exon 12 mutations, *MPL*W515 mutations, and *CALR* indels, covering virtually all PV cases (~99%) and the vast majority of ET and PMF cases (85–90%), and less common *non-driver* mutations, including *TET2*, *ASXL1*, *IDH1/2*, *EZH2*, *SRSF2*, *LNK*, *CBL*, *TP53*, etc., occurring in fewer than 30% of patients during chronic phase but typically increasing with disease progression (accelerated/blast phase) [2].

These mutations represent crucial molecular events in the pathogenesis of MPNs, affecting disease phenotype, course, and outcome [3], and have, therefore, been included into WHO diagnostic criteria as well as in the “genetically inspired” prognostic scoring systems for disease risk stratification (i.e., the Mutation-enhanced International Prognostic Scoring System for transplant-age patients (MIPSS70), the karyotype-enhanced MIPSS70 (MIPSS70 ver2.0), and the Genetically inspired Prognostic Scoring System) (GIPSS) [4,5,6].

However, data from epidemiological and familial studies clearly point out a heritable component affecting the risk of developing MPN and potentially contributing to the phenotypic pleiotropy observed despite shared driver mutations [7].

Hereditary predisposition to MPNs usually refers to common, low-penetrance, germline host genetic factors, detected in the general population, whose presence facilitates the acquisition of a somatic driver mutation in a pluripotent hematopoietic stem cell, giving rise to the malignant clone [8].

Genome-wide association studies have identified a number of germline genetic patterns associated to an increased propensity for developing sporadic MPNs [9,10,11,12,13]. In addition, a small number of genetic variants are associated with familial predisposition. In these cases, a germline variant has been recurrently identified in family clusters of MPNs [7].

In this review we covered the main host genetic variants that have been associated to familial and sporadic MPN in terms of (1) increased disease risk, (2) impact on disease phenotype and outcome, and (3) influence on therapy response, discussing them in the functional perspective of the biological pathways involved.

## 2. Host Genetic Variants Associated to Familial MPNs

Host genetic factors in familial MPNs include germline duplication of *ATG2B* and *GSKIP* [14], germline *RBBP6* mutations [15], and germline variants in *LRRC3* and *BCORL1* [16]. Of note, these MPNs do not phenotypically differ from sporadic cases and, interestingly, the affected members may carry different somatic driver mutations (*JAK2*V617F, *MPL*, and *CALR*) [7,8]. MPN phenotype encompasses PV, ET, and PMF in the first two studies [14,15] and only PV in the paper by Hirvonen et al. [16]. In this latter case, all patients displayed *JAK2*V617F mutation.

It has been hypothesized that overexpression of *ATG2B* and *GSKI*, resulting from germline duplication, may account for increased fitness for cells subsequently acquiring somatic mutation(s), hinting, therefore, that germline variants and acquired somatic driver mutations are cooperative events for MPN development [14].

*RBBP6* gene encodes for a ring finger E3 ubiquitin ligase involved in p53 degradation. *RBBP*R1569H germline variant alters the p53 binding site, thus conferring an increased risk of mutagenesis. In this case, therefore, the correlation with all three hallmark driver mutations is explained by genetic instability [15].

## 3. Host Genetic Variants Associated to Increased MPN Risk in General Population

Germline variants reaching the conventional threshold (*p* < 5 × 10^−8^) for genome-wide significant association with an increased MPN risk are summarized in Table 1.

Germline polymorphisms at *JAK2* and *TERT* endow individuals with a predisposition to developing a MPN, which emerged from all the four main studies interrogating large patient and control data sets by a genome-wide association approach [10,11,12,13]. Polymorphisms at 3q26.2 and 3q26.3, involving *MECOM* and the intergenic region between *HBS1L* and *MYB*, have been associated to increased MPN risk in three out of the four above referenced studies [11,12,13]; the association did not emerge in the Icelandic population, described by Oddsson et al. [10].

Other recurrent variants include *TET2*, *ATM*, *SH2B3*, *PINT*, *CHECK2*, and *GFI1B* loci, first identified by Hind et al. [11] and very recently confirmed by Bao and colleagues [13].

### 3.1. The JAK2 46/1 Haplotype

The *JAK2* “46/1” or “GGCC” haplotype has been the first germline risk variant described in MPNs, and simultaneously reported by three independent groups of investigators in 2009 [9,17,18]. The *JAK2* 46/1 haplotype maps on a region of about 250–280 Kb on the short arm of chromosome 9 including, in addition to *JAK2*, also Insulin-like 6 (*INSL6*) and *INSL4* genes. The haplotype is tagged by a combination of four single nucleotide polymorphisms (SNPs)—rs3780367, rs10974944, rs12343867, and rs1159782—mapping on *JAK2* introns 10, 12, 14, and 15 and generating the so-called “GGCC” sequence.

These SNPs are in complete linkage disequilibrium and, therefore, inherited en bloc. The frequency of this haplotype is around 45% in the general population and its presence has been associated to an increased risk of MPN onset, preferentially—but not exclusively—carrying *JAK2* driver mutations (including V617F exon 14 mutation and exon 12 mutations). This is likely due to an increased susceptibility to DNA damage and replication errors conferred by the haplotype, which may predispose to the acquisition of the somatic *JAK2* mutations (“hypermutability hypothesis”) or, at the opposite, may confer a selective advantage to randomly *JAK2*-mutated clone (“fertile ground hypothesis”) [17].

Although the association is stronger with *JAK2*V617F-positive MPNs, recent GWAS studies demonstrate that the *JAK2* 46/1 haplotype reaches the genome-wide level of significance (*p* < 5 × 10^−8^) also in *JAK2*V617F-negative cases [12] including those carrying *CALR* mutations [18]. These data, of course, should lead us to search for a broader explanation of the mechanisms by which the *JAK2* 46/1 haplotype predisposes to MPN, which likely goes beyond the “hypermutability” or “fertile ground” hypotheses, which can explain the association with *JAK2*V617-positive but not with *JAK2*V617F-negative cases (i.e., increased mutation rate of the *JAK2* locus and selective advantage of the *JAK2*V617F mutated clone) [18].

The demonstration of broader, pleiotropic effects of these germline variants derives from a study conducted in patients with normal karyotype acute myeloid leukemia, showing that homozygosity or heterozygosity for the *JAK2* 46/1 haplotype was associated with myelomonocytic-biases malignant hematopoiesis and increased risk of death from infection due to an aberrant immune response [19].

In this regard, an intriguing perspective was provided by Hermouet and coll [20], who hypothesized that the 46/1 haplotype may facilitate the overexpression of the *JAK2* gene (by recombination or abnormal promoter binding/methylation) and the consequent activation of pro-proliferative and pro-survival pathways in myeloid cells, which, in turn, predisposes to an increased risk of DNA replication errors and mutations in pathogenetically relevant myeloid genes (including *CALR*, *MPL*, *TET2*, *ASXL1*, etc.). This would explain the association with *JAK2*-unmutated MPNs. Moreover, the overexpression of *INSL4* and *INSL6* (also part of the haplotype) in bone marrow stroma cells may lead to an increased production of pro-inflammatory cytokines, generating a permissive milieu for the mutated clone [20]. Overall, the *JAK2* 46/1 haplotype could be envisioned as a host genetic susceptibility factor for inappropriate myeloid response to cytokines, leading to an enhanced inflammatory state and increased risk of myeloid neoplasms.

### 3.2. Telomere Reverse Transcriptase Gene (TERT) Polymorphisms

The telomere reverse transcriptase gene (*TERT*) encodes for the catalytic component of telomerase, a ribonucleoprotein enzyme that stabilizes telomere length, preventing the activation of the cellular senescence program [21]. *TERT* biological function makes it a strong candidate for factors that influence cancer risk. Indeed, enhanced telomerase activity/expression has been extensively associated to several types of cancers [22]. The impact of short telomeres in disease was first appreciated in dyskeratosis congenita (DC), a disorder typified by the mucocutaneous triad (oral leukoplakia, abnormal skin pigmentation, and nail dystrophy), bone marrow failure, cancer predisposition, pulmonary fibrosis, and liver disfunction [23]. The high penetrance of bone marrow failure among patients with classic DC highlights the importance of telomere length maintenance in hematopoiesis. Indeed, the mechanism underlying DC-related bone marrow failure appears to be related to a progressive depletion of functional hematopoietic progenitor and stem cells [24,25].

The rs2736100 SNP located in the second intron of the *TERT* gene at 5p15 has been associated to an increased risk of cancer [26], including MPNs. According to Tapper et al. [12], the association between the rs2736100 SNP and MPN risk reaches, however, genome- wide significance only when including *JAK2*V617F-positive cases. Hence, similarly to the JAK2 46/1 haplotype, the effects are stronger in *JAK2*V617F-mutated MPNs. According to Trifa et al. [18], the SNP was significantly associated with each single MPN, regardless of driver-mutation status.

This SNP was first described by Oddsson et al. [10] in 237 Icelanders diagnosed with MPN and, although the underlying functional mechanism is still debated, it is thought to (1) directly increase the transcription of TERT, enhancing its expression, and (2) be in linkage disequilibrium with biologically plausible disease-causing mutations.

In addition to the rs2736100, other variants are enriched in MPN populations, specifically the rs7705526 and the rs2853677 [11,13], which, similarly to the rs2736100, emerged from previous GWAS cancer studies [27]. Hind et al. showed that the lead SNP rs2853677 is in moderate linkage disequilibrium with the rs2736100 [11].

Interestingly, a growing body of evidence has been accumulated on non-canonical functions of telomerase reverse transcriptase, including cell cycle regulation, promotion of cell growth and proliferation, and control of mitochondrial integrity following oxidative stress [28]. Wang et al. observed a dose-dependent effect of the rs2736100 G-allele on IL-6 levels in non-small cell lung cancer, suggesting a role for this SNP in *IL6* gene expression modulation and cytokine production [29]. This observation is of utmost interest in the context of MPNs, in which the inflammatory background is the main trigger and driver for clonal evolution [30].

Besides *TERT* rs2736100, telomere length is genetically determined by, at least, 10 other SNPs (*ZNF676* rs412658, *CTC1* rs3027234, *DHX35* rs6028466, *PXK* rs6772228, *NAF1* rs7675998, *ZNF208* rs8105767, *OBFC1* rs9420907, *ACYP2* rs11125529, *TERC* rs10936599, and *ZBTB46* rs755017) [31,32,33,34]. This observation derives from studies on telomere length measured in leukocytes (LTL), which is considered a reliable surrogate for telomere length in other tissues, relatively stable over time. A polygenic risk score (named “teloscore”) based on the combination of these 11 SNPs—detected by GWAS—has been successfully utilized to investigate the association between genetic determinants of telomere length and increased cancer risk.

Giaccherini et al. [35] tested the association between teloscore (built by weighting the effects of each of the 11 SNPs on LTL) and MPN risk, surprisingly finding that genetically determined longer telomeres predict higher MPN risk. Individual SNP association studies (by allelic discrimination assays) confirmed the *TERT* rs2736100 C-allele as a high-risk variant for increased MPN susceptibility and additionally reported a novel association of the *OBFC1* rs9420907 C allele and increased MPN risk.

### 3.3. Polymorphisms in3q.26 (MECOM and HBS1L-MYB)

Polymorphisms at 3q26.2 and 3q26.3, involving *MECOM* and the intergenic region between *HBS1L* and *MYB* (the so-called HMIP region), account for increased MPN risk.

*MECOM* (*MDS1 and EVI1 Complex Locus*) gene encodes for a transcription factor involved in hematopoietic stem cell maintenance, differentiation, and leukemogenesis [36]. *MECOM* has a well-established role in hematologic neoplasms. Germline mutations have been reported to be causative of a rare, inherited bone marrow failure syndrome with a megakaryocytic thrombocytopenia associated to various organ malformations with variable penetrance [37]. In addition, chromosomal rearrangements between 3q21 and 3q26 elicit high-risk acute myeloid leukemia, via the *GATA2* enhancer reposition near the *MECOM* locus, which results in both *EVI1* overexpression and *GATA2* haploinsufficiency [38,39]

Three different *MECOM* SNPs have been identified as genome-wide significant loci in MPNs: the rs22018862 [12], the rs3851397 [11], and the rs9847631 [13]. In the study authored by Tapper et al. [12], the rs22018862 SNP, located in a non-coding region 153 Kb downstream of *MECOM*, was the only germline variant that, along with the *JAK2* 46/1 haplotype, maintained genome-wide significance when analyses were restricted to *JAK2*V617F-negative MPNs. Functional implications of this SNP are not established yet; however, based on publicly available databases, the authors hypothesized that this region may correspond to a regulatory element with enhancer-like activity. The rs22018862 SNP appears to correlate with PV and *CALR*-mutated ET and PMF [18].

*HMIP* SNPs, located in the intergenic region between *HBS1L* and *MYB*, account for interindividual variability of hematologic parameters in healthy subjects, particularly of platelet, monocyte, and erythrocyte counts [40,41,42,43,44]. Additionally, polymorphisms of the *HBS1L-MYB* intergenic region have been reported to influence fetal hemoglobin levels in adults [45], with relevant functional and clinical implications in β hemoglobinopathies [46,47]. The mechanism by which these noncoding sequence variants affect multiple erythrocyte characteristics has been elucidated in subsequent, follow-up studies, demonstrating that *HMIP* polymorphisms affect regulatory elements bound by erythroid transcription factors. These elements interact with *MYB*, a critical regulator of erythroid development and HbF levels [48]. In fact, it has been shown that *MYB* plays crucial role in silencing the fetal and embryonic hemoglobin genes [49].

Two GWAS studies identified the rs9376092 SNP as a susceptibility germline variant for increased MPN risk [11,12]. The rs9376092 is in strong linkage disequilibrium with the other SNPs tagging the *HMIP* region. The polymorphic allele variant modulates the expression of both flanking genes, especially of *MYB*, which is down-modulated. Interestingly, *MYB* down-regulation is responsible for enhanced normal and clonal megakaryopoiesis, as demonstrated by the fact that *MYB* knockdown in normal human hematopoietic progenitors promotes megakaryocyte development [50], and that *MYB* knockdown in the murine Kit+Sca1+Lin- hematopoietic stem cell population generates a transplantable myeloproliferative phenotype that mimics ET [51].

Consistently, genotype–phenotype correlations by Trifa and Colleagues indicated that the rs9376092 SNP preferentially associates with *JAK2*V617F-positive ET [18].

Of note, *MYB* deregulation from a novel *EWSR1-MYB* fusion was detected at the time of leukemic evolution of a *JAK2*V617F-positive PMF case [52].

### 3.4. GFI1B and CHEK2 Polymorphisms

*GFI1B* and *CHEK2* SNPs were first reported as genome-wide significant loci for increased MPN risk by Hind et al. [11]. Very recently, Bao and Colleagues provided a complete functional characterization of the mechanisms by which *GFI1B* and *CHEK2* genetic variations affect the MPN risk through the modulation of hematopoietic stem cell function [13].

*GFI1B* mutations have been associated to a rare, dominant, congenital platelet disorder known as *GFI1B*-related thrombocytopenia (*GFI1B*-RT), caused by the presence of truncated *GFI1B* proteins with dominant-negative properties on megakaryocytopoiesis and thrombopoiesis [53]. The SNPs accounting for MPN predisposition are the rs621940 [11], the rs1633768, and rs524137 [13], of which the last two are located in a region of hematopoietic-accessible chromatin located around 12 kb downstream of *GFI1B*.

The checkpoint kinase *CHEK2* is a critical component of the DNA damage response pathway. Germline variants reaching GWAS-significant association with increased MPN risk include the rs555607708 [11] and the I157T missense variant (rs17879961) [13], which have been previously linked to an increased risk for several types of cancer, including chronic lymphocytic leukemia [54].

Bao and coll. provided the first demonstration that the rs524137 SNP of *GFI1B* and the rs17879961 SNP of *CHEK2* are functionally relevant, by reducing, respectively, *GFI1B* expression and *CHEK2* function in hematopoietic stem cell and thereby increasing their self-renewal [13].

## 4. Host Genetic Variants Modulating Disease Phenotype and/or Outcome

Although not implicated in conferring an increased MPN risk, several SNPs have been described to harbor disease-modifying effects, in terms of phenotype, hematologic parameters at the time of MPN onset, disease course, and outcome. Moreover, the *JAK2* haplotype, repeatedly confirmed as a strong host genetic predisposition factor for MPN in GWAS, has been identified as a biomarker of disease outcome in PMF by Tefferi’s group. In a first study published in 2010, nullizygosity for the *JAK2* haplotype was associated to shortened survival in a cohort of 130 PMF patients [55]. Subsequently, in a follow-up study on a cohort of 414 molecularly annotated PMF, the authors confirmed that wild-type patients displayed inferior overall survival as compared to the other genotypes, independently from well-established genetic and cytogenetic markers of poor outcome (karyotype, driver mutational status, presence of high-molecular-risk mutations) [56].

Table 2 summarizes SNPs capable to influence MPN phenotype and outcome, whose early detection is shaping as an informative tool for personalized patient follow-up and treatment planning.

### 4.1. The rs6198 SNP of the Glucocorticoid Gene

The human glucocorticoid receptor is encoded by *NR3C1* located in the 5q31-32 cytoband of chromosome 5 and is composed of nine exons with five splicing variants: GRα, GRβ, GRγ, GR-A, and GR-P. GRα and GRβ are generated by alternative splicing of exons 9α and 9β, respectively. While GRα resides primarily in the cytoplasm and can interact with endogenous or synthetic agonists, GRβ constitutively resides in the nucleus, where it controls transcription primarily by a dominant-negative effect on GRα-induced gene expression [62].

The *NR3C1* gene is highly polymorphic. The rs6198 A to G SNP is located within the 3′ untranslated region and it increases the stability of GRβ mRNA, with a half-life up to ≥ 6 h, enhancing, therefore, GRβ expression [63]. In healthy subjects, this SNP is present with an allele frequency between 4% (sub-Saharan Africans) and 20% (Europeans) but its frequency increases in patients with autoimmune disorders, where it also mediates glucocorticoid resistance [64].

The biological role of GRβ and its SNP in MPNs were first investigated by Varricchio and coworkers [57]. The authors found that the dominant negative β isoform of the glucocorticoid receptor is selectively expressed in erythroid cells expanded from patients with polycythemia vera, where it contributes to the development of erythrocytosis. GRβ isoform expression is likely attributable to the increased prevalence of the rs6198 SNP of *GR* in PV patients, reporting a 55% frequency of the polymorphic allele variant as compared to 9% in healthy control subjects. Given this background, the same group analyzed frequency, genotype–phenotype correlations, and impact on disease progression of the rs6198 SNP of *GR* in a cohort of 499 PMF. The rs6198 variant, as tagged by the G allele, occurred with a higher frequency in PMF patients as compared to 111 local healthy volunteers and 2837 controls from the UK (genotyped by the WTCCC). Homozygosity for the SNP is associated with higher white blood cell count, splenomegaly, and higher circulating CD34+ cells at the time of diagnosis. Finally, *JAK2*V617F-positive PMF carrying both risk alleles display shorter overall and leukemia-free survival as compared to the other genotypes [58]. Therefore, the rs6198 SNP should be considered a host genetic factor for adverse presentation and outcome (when associated to the *JAK2*V617F mutation) in PMF.

### 4.2. The rs1024611 SNP of CCL2

The *CCL2* gene, encoding for the chemokine CCL2, also known as Monocyte Chemoattractant Protein-1 (MCP-1), is located in the q11.2-12 cytoband of chromosome 17 and, similarly to other cytokine genes, is highly polymorphic. The rs1024611 SNP, characterized by an A to G substitution in the distal regulatory region of the gene, modulates the transcriptional activity of *CCL2*, accounting, therefore, for interindividual variability in the levels of circulating chemokine. As a result, G/G homozygous individuals are the highest chemokine producers. In a healthy population, the G allele is over-represented in Asians and Mexicans as compared to Caucasians and African Americans [65,66]. Subjects carrying the rs1024611 SNP of *CCL2* display an increased susceptibility to several conditions such as autoimmune disorders, atherosclerosis, and cancer [67,68,69].

We investigated the prevalence of the rs1024611 SNP of *CCL2* in MPNs, finding similar genotypic and allelic frequencies among PV, ET, and control subjects. Focusing on MF, which can be considered the MPN variant characterized by the highest inflammation burden, we found that postPV/ET MF were significantly enriched in polymorphic individuals as compared to (1) overall PMF, (2) prePMF, (3) overtPMF patients, and (4) healthy controls. Additionally, in MF patients, genotype–phenotype correlation studies pointed out a higher frequency of allele-G carriers in (1) intermediate-2/high-IPSS-risk group; (2) patients with severe anemia (Hb < 100 g/L); (3) patients with ≥1% circulating blasts; and (4) patients with ≥ II grading of bone marrow fibrosis [59].

Recently, in a cohort of 773 PMF, we assessed the contribution of this SNP in terms of increased disease risk and its potential effect on disease outcome, demonstrating that male subjects carrying the homozygous genotype G/G had an increased risk of PMF, hinting at a potential genetic interplay between gender and the G-allele variant. We also found that G/G PMF displayed a significantly reduced survival. In combined analysis of the G/G genotype with other well-established clinical prognostic parameters included in the IPSS scoring system we found a significant correlation in both univariate and multivariate analysis. Finally, we determined the functional implication of this SNP in PMF, showing that higher CCL2 circulating levels, determined by the G/G rs1024611 genotype, are biologically relevant since PMF hematopoietic progenitors selectively express CCR2 and activate a pro-survival, Akt-dependent signaling pathway when stimulated with CCL2 [60].

### 4.3. The rs2431697 of MIR146A

NF-κB signaling hyperactivation has been described in mouse models of MPN as well as in MF and MPN blast phase [70]. Indeed, NF-κB has been identified as a key mediator of inflammation-triggered carcinogenesis [71] and, in MF, it has been suggested to cooperate with JAK2 signaling hyperactivation in supporting cytokine overproduction [70]. On the other side, NF-κB itself mediates the signal transduction downstream of several cytokine and chemokine receptors, including CCR2 [72], which plays a relevant role in PMF pathophysiology, as above described.

NF-κB is regulated by microRNAs (miRNAs), small, single-stranded non-coding RNA molecules capable to regulate gene expression. The miR-146a counteracts the NF-κB-dependent proinflammatory signal via inhibition of TNF Receptor-Associated Factor 6 (TRAF6) and Interleukin 1 Receptor-Associated Kinase 1 (IRAK1). Indeed, miR-146a deficiency accounts for a chronic inflammatory phenotype and enhanced myeloproliferation [73].

MiR-146a levels are regulated by two functional SNPs: rs2910164 and rs2431697, the latter accounting for lower miR-146a expression. Ferrer-Marin et al. investigated whether these SNPs could represent susceptibility factors for MPNs, demonstrating—in a cohort of 967 MPNs—that the rs2431697 T/T genotype (1) is associated to increased pro-inflammatory cytokine levels in MPN patients; (2) is enriched in post ET/PV MF; and (3) independently predicts a shorter time to MF progression in PV and ET. These data further support the well-consolidated concept of a “biological continuum” from PV/ET (early-stage diseases with a relatively milder phenotype) to secondary MF (end-stage disease), which parallels a progressive increase in the inflammatory burden [61].

## 5. Host Genetic Variants Affecting Therapy Response

All four type-III *IFNs* (*IFN-λ1*, *2*, *3*, and *4*) are encoded by genes located within a ~55-kb genomic region on human chromosome 19, which is highly polymorphic. Genetic variants within the *IFNL* region have been reported to strongly influence both spontaneous and INF-α-induced clearance of hepatitis C virus infection [74].

The INF-α-based regimen is capable to induce durable hematologic response in MPN patients, and, by significantly lowering the *JAK2*V617F allele burden, proved to be able to effectively reduce the size of the malignant clone [75].

SNPs located near the *IL28B* gene, encoding for interferon-λ 3 (*IFNL3*), and in the *IFNL4* gene encoding for interferon-λ 4 were recently associated to an INF-α-based regimen response in PV patients (Table 3).

Lindgren and Colleagues, by retrospectively analyzing a cohort of 100 MPN patients (47 PVs, 43 ETs, and 10 MFs), demonstrated that the homozygosity (C/C genotype) for the rs12979860 located in between *IFNL3* and *IFNL4* was significantly associated with achievement of complete hematologic response (HR) in PVs (79% in C/C patients vs. 48% of other genotypes) [76].

In line with these findings, Jager et al. evaluated how germline variants of *INFL3*/*INFL4* loci influenced the hematologic and molecular response (MR) in a cohort of 122 PV patients enrolled in the PROUD-PV and CONTINUATION-PV trials treated with ropeginterferon alfa-2b, over a follow-up period of 36 months. An initial GWAS screening analysis did not reveal any significant associations (*p* < 5 × 10^−8^) between germline genetic variants and the achievement of HR and MR. However, target association analyses, focused on rs8099917, rs12979860, rs368234815, and rs117648444, showed that rs8099917 T/T, rs12979860 C/C, and rs368234815 TT/TT genotypes individually correlated with higher MR rates. In addition, functional diplotypes affecting INF4 production were associated with variable MR rates, with haplotype pairs determining loss of function/impaired function protein variants being associated with higher responses [77].

Therefore, functional *INFL* locus SNPs may explain heterogeneity in INF-α response in MPN patients.

## 6. Host Genetic Determinants of Clonal Hematopoiesis of Indeterminate Potential (CHIP)

CHIP refers to an age-related phenomenon characterized by the acquisition of somatic mutations leading to the expansion of clonal hematopoietic stem and progenitor cells. A relevant clinical scenario associated with CHIP is represented by the increased risk of cardiovascular events, likely driven by an increased inflammatory state sustained by mature, mutant myeloid cells [78].

The two most commonly mutated genes in CHIP are *DNMT3A* and *TET2*, followed by *ASXL1*, splicing factors, *JAK2*, and *TP53* [78]. In addition to acquired somatic mutations, two recent genome-wide association studies identified germline genetic variants that predispose to CHIP [79,80].

By whole-genome sequencing on 11.262 Icelanders, Zink et al. [79] described a germline deletion located in intron 3 of *TERT* (rs34002450) that was significantly associated to CHIP. This finding hinted to a role of telomerase activity in CHIP, both age-related phenomena. Indeed, the Authors demonstrated that telomere length estimates were significantly lower in people with CHIP [79].

Very recently, Bick et al. [80] identified three genome-wide significant loci conferring an increased risk of CHIP involving *TERT*, *TET2*, and the intergenic region spanning *KPNA4-TRIM59*. Concerning *TERT*, the authors replicated the association with rs34002450 described by Zink. et al. [79] and additionally identified a second intronic *TERT* variant—rs13167280—independently associated with CHIP status.

*TET*2 variant, namely, rs1444188061, was found exclusively in samples from individuals with African ancestry, and, despite selectively involving *TET2* gene, it was equally robustly associated with *DNMT3A*-, *TET2*-, and *ASXL1*-dependent CHIP; this has been ascribed to the fact that *TET2* risk variant disrupts the distal enhancer of the gene, hampering *TET2* expression, therefore increasing self-renewal of hematopoietic stem cells [80].

Overall, these seminal studies indicate that germline variants not only affect the risk of developing a hematologic malignancy, but also intervene in shaping the risk of early, pre-malignant states such as CHIP. Of note, both *TERT* and *KPNA4* display germline variants predisposed to CHIP [79,80] and increased MPN risk [13].

## 7. Conclusions and Perspectives

Germline genetic factors play a relevant role in determining individual predisposition to develop an MPN and, once the disease is acquired, in affecting disease presentation, course, therapy response, and outcome. This is consistent with the fact that disorders sharing the same somatic driver mutations are nevertheless typified by an extremely heterogenous presentation and behavior. The increasingly widespread use of novel techniques such as Next-Generation Sequencing during the routine diagnostic work-up of MPN patients will allow us to detect prognostically relevant SNPs and potentially include them in a personalized risk stratification, as envisioned, for example, for the *JAK2* 46/1 haplotype [81].

Indeed, the use of SNP arrays with custom probes for selected, informative gene variants may lead in the future to a germline genetic screening for MPN patients, aiming to identify those being more likely to experience an unfavorable disease course and poor response to therapy, and providing, therefore, physicians with novel tools for adequate patient counseling and personalized disease monitoring.

**Figure 1:**
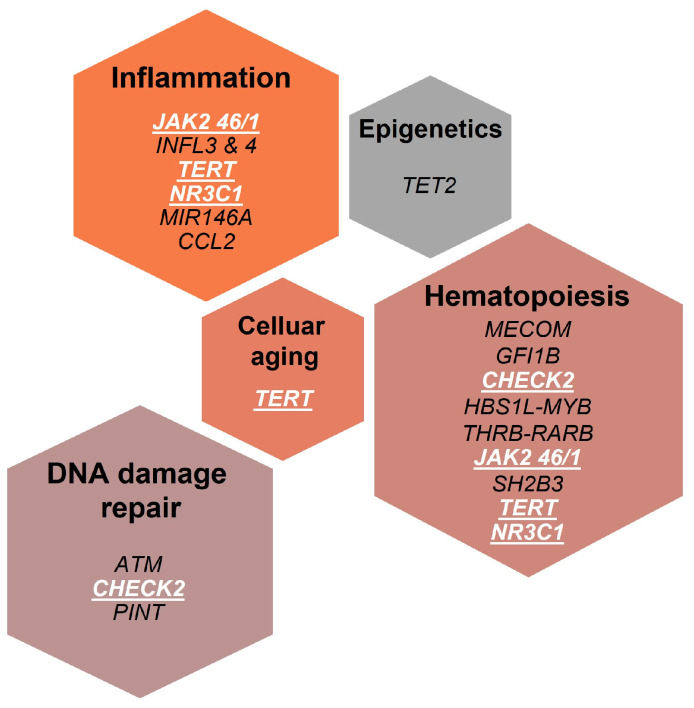
Germline variants associated with MPN risk, phenotype, outcome, and therapy response classified according to their biological function. Genes exerting multiple functions are highlighted in white.

Clinically relevant SNPs in MPNs discussed in this review basically affect genes involved in the three main biological processes: (1) hematopoiesis; (2) DNA damage repair; and (3) inflammation (Figure 1):

(1) Hematopoiesis defines the tightly regulated process of formation of blood and immune cells. To generate these cells, HSCs give rise—throughout individuals’ life span—to an array of committed progenitors, which proliferate extensively and then differentiate into mature cells. Recent advances in genomics, such as accurate deep sequencing and novel methods of cell tracking, revolutionized the concept of hematopoiesis from a process made of discrete, punctuated phenotypic changes to a “continuum model”, typified by a continuous process of differentiation with blurred demarcation between different stages [82,83]. Genetic studies revealed, also, how mechanisms underlying hematopoiesis are modulated by genetic variations present throughout the population. The importance of these host genetic variations is highlighted by the fact that clinically measured hematopoietic traits typically show extensive interindividual variability and are highly heritable, which means that a relevant part of the observed phenotype variations can be attributed to genetic factors [82,84].
(2) During genome duplication, cells may experience different exogenous and endogenous replication stresses, hampering the progression of DNA replication. Replication stress is a phenomenon exacerbated in cancer cells because of the loss of DNA repair genes or the activation of oncogenic pathways [85]. To counteract replication stress, cells are equipped with DNA damage response, an extensive network of signaling pathways accounting for recognition of DNA damage, DNA remodeling and repair, DNA damage bypass during replication, cell cycle control, and cell fate decisions in response to DNA alterations [86]. More than 450 genes are involved in this network. In addition to MPNs, a variety of polymorphisms in *DDR* genes have been associated with increased risk of developing acute myeloid leukemia [87] and breast cancer [88].
(3) Inflammation refers to a host defense mechanism orchestrated by the immune system in response to harmful stimuli, such as pathogens, damaged cells, toxic compounds, or irradiation [89]. Cytokines are key mediators of the inflammatory response, by promoting the recruitment and activation of immune cells. After the human leucocyte antigen (*HLA*), chemokine genes are probably one of the most polymorphic sets of genes in the immune system, with remarkable effects on the immune response. A number of functionally relevant cytokine SNPs have been found repeatedly associated with disease of different etiologies but sharing a common pathogenetic aspect such as chronic inflammation [67].

Concerning the functional overview of MPN-related SNPs reported in Figure 1, we can observe that some of the genes involved have multiple functions, with consequent overlap between categories. Moreover, in addition to the above-described overarching categories (hematopoiesis, DNA damage repair, and inflammation), *TERT* (involved in the cellular aging process) and *TET2* (regulating epigenetic changes) must be considered as well.

Examples of functional overlap involve the *JAK2* haplotype, playing a role not only in hematopoietic stem/progenitor cell fate by predisposing to the acquisition of autonomous cell growth [9,17,18] but also in systemic inflammation, by fueling an inappropriate myeloid response to cytokines [20]. Similarly, the *NR3C1* SNP predisposes to both immune dysregulation—by mediating glucocorticoid resistance [90]—and to JAK/STAT signaling abnormalities by associating with STAT5 [57]. Additionally, *TERT* gene not only regulates cell senescence but also influences *IL6* gene expression and hematopoiesis [29]. Finally, *CHEK2* gene, a well-characterized component of the DNA damage response pathway, has been endowed with novel regulatory functions in determining hematopoietic stem cell fate [13].

Overall, most of these MPN SNPs share a role in modulating the individual proinflammatory state, being the common host genetic denominator of immune dysregulation, chronic inflammation, and clonal proliferation.

MPNs are a well-established paradigm of oncoinflammatory disorders [30], and it is reasonable to hypothesize that the genetically determined host inflammatory background exerts a relevant role in influencing the features of the disease, thus accounting for phenotypic diversity in MPNs.

## Figures and Tables

**Table 1:** Genome-wide significant host genetic variants defining the MPN risk.

SNPs	Gene Function (Relative to Hematopoiesis)	Associated Driver Mutations	Associated MPN Phenotype	Ref.
***JAK2*46/1** haplotype	Hematopoiesis, cytokine receptor signaling	All (>*JAK2*V617F)	All (>PV and PMF)	[9,10,11,12,13]
***TERT***	Telomere length	All	All	
rs2736100				[10,12]
rs7705526				[11,13]
rs2853677				[11,13]
***MECOM***	HSC maintenance, differentiation	*JAK2*V617F and *CALR* type 1/type 1-like	PV MF and ET (only in presence of *CALR*)	
rs2201862				[12]
rs3851397				[11]
rs9847631				[13]
***HBS1L-MYB*** rs9376092	Peripheral blood cell counts, fetal hemoglobin levels	none	ET (only in presence of *JAK2*V617F)	[11,12]
***GFI1B***	HSC quiescence, erythroid and megakaryocytic differentiation	n/a	n/a	
rs621940				[11]
rs1633768				[13]
rs524137				[13]
***CHEK2***	DNA damage response	n/a	n/a	
rs555607708				[11]
rs17879961				[13]
***SH2B3*** rs7310615	Negative regulation of normal hematopoiesis	n/a	n/a	[11,13]
***ATM*** rs1800057	DNA damage response	n/a	n/a	[11,13]
***TET2***	HSC self-renewal, commitment, terminal differentiation of monocytes	n/a	n/a	
rs1548483				[11]
rs62329718				[13]
***PINT*** rs58270997	DNA damage response, hematopoietic stem cell maintenance, and differentiation (via PRC2)	n/a	n/a	[11,13]
***THRB-RARB*** rs4858647	unknown	none	PMF	[12]
***GATA2*** rs9864772	HSC activity and self-renewal, myeloid and myelo-erythroid differentiation, erythroid precursors maintenance	n/a	n/a	[13]
***SCHIP1*** rs77249081	unknown	n/a	n/a	[13]
***KPNA4*** rs74676712	unknown	n/a	n/a	[13]
***NUDT3*** rs116466979	unknown	n/a	n/a	[13]
***MKLN1*** rs61471615	unknown	n/a	n/a	[13]
***MRPS31*** rs8002412	unknown	n/a	n/a	[13]
***ZNF521*** rs9946154	HSC differentiation and B-lymphoid cell development	n/a	n/a	[13]
***RUNX1*** rs55857134	Differentiation of megakaryocytes and lymphocytes	n/a	n/a	[13]

Abbreviations: HSC: Hematopoietic Stem Cell; n/a: not assessed; PRC2: polycomb repressive complex 2; > indicates a stronger association.

**Table 2:** Host genetic variants affecting MPN phenotype and/or outcome.

SNPs	Gene Function (Relative to Hematopoiesis)	Detection Methods	Allele Variant	MPN Cohort	Disease Subtype Associations	Disease Phenotype Associations	Ref.
***JAK2* 46/1 haplotype** **rs12343867** **(T/C)**	Hematopoiesis, cytokine receptor signaling	RT-PCR	T allele (wild type)	130 PMF	n/e	↓ OS	[55]
		RT-PCR	T allele (wild type)	414 PMF	n/e	↓ OS	[56]
***NR3C1* rs6198** **(A/G)**	Immune response regulation, erythrocytosis	PCR-SSCP + sequencing	G-allele	57 MPNs 22 CTRLs	PV	n/e	[57]
		HRM analysis + sequencing	G-allele (homozygous)	499 PMF 2948 CTRLs	PMF	↑ CD34+ cells, splenomegaly, ↑WBC, ↓ LFS *	[58]
***CCL2*** **rs1024611** **(A/G)**	Chemokine production	RT-PCR	G-allele	177 MPNs 149 CTRLs	sMF	↓ Hb, ↑ IPSS, ↑ blasts, ↑ fibrosis	[59]
		RT-PCR	G-allele (homozygous)	773 PMF 323 CTRLs	PMF in males	↓ OS	[60]
***MIR146A* rs2431697** **(C/T)**	NF-κB signaling modulation	RT-PCR	T-allele (homozygous)	967 MPNs 600 CTRLs	sMF	↓ MF-free survival in PV and ET	[61]

Abbreviations: RT-PCR: real-time Polymerase Chain Reaction; OS: Overall Survival; PCR-SSCP: Polymerase chain reaction-single-stranded conformation polymorphism; HRM: High-Resolution Melting; n/e: not evaluated; WBC: White Blood Cells; LFS: Leukemia-free survival; Hb: hemoglobin; IPSS: International Prognostic Scoring System. * only for *JAK2*V617F^pos^ PMF.

**Table 3:** Host genetic variants affecting response to therapy in MPNs.

SNPs	Gene Function (Relative to Hematopoiesis)	Detection Methods	Allele Variant	MPN Cohort	Type of Therapy	Response Assessment	Association(s)	Ref.
***INFL4*** **rs12979860** **(T/C)**	Cytokine production	RT-PCR	C/C	100 MPNs	Inf α-2b, peg α-2b peg α-2a	HR	Higher HR rate in PVs	[76]
		RT-PCR	C/C	122 PVs	ropeg α-2b	HR, MR	Higher MR rate	[77]
***INFL4*** **rs368234815 (G/TT)**		RT-PCR	TT/TT	122 PVs	ropeg α-2b	HR, MR	Higher MR rate	[77]
***INFL4*** **rs8099917** **(T/G)**		RT-PCR	T/T					

Abbreviations: RT-PCR: real-time Polymerase Chain Reaction; HR: Hematologic Response; Inf: interferon; MR: Molecular Response; peg α-2b/-2a: peginterferon α-2b/-2a; ropeg α-2b: ropeginterferon α-2b.
